# Transcriptomic Characterization of Cellular Senescence in Human Donor Livers Before Transplantation

**DOI:** 10.1111/acel.70828

**Published:** 2026-11-11

**Authors:** Adam M. Thorne, Sabrina Stimmeder, Kirill Ustyantsev, Niels Mulder, Laura Niedernhofer, Ton Lisman, Marco Demaria, Eugene Berezikov, Folkert Kuipers, Vincent E. de Meijer

**Affiliations:** ^1^ Department of Liver Transplantation and HPB Surgery University of Groningen, University Medical Center Groningen Groningen the Netherlands; ^2^ UMCG Comprehensive Transplant Center Groningen the Netherlands; ^3^ Department of Surgery, Division of General, Visceral and Transplant Surgery Medical University of Graz Graz Austria; ^4^ European Research Institute for the Biology of Ageing (ERIBA) University of Groningen, University Medical Center Groningen Groningen the Netherlands; ^5^ Department of Pediatrics University of Groningen, University Medical Center Groningen Groningen the Netherlands; ^6^ Masonic Institute on the Biology of Aging and Metabolism University of Minnesota Minneapolis Minnesota USA; ^7^ Department of Biochemistry, Molecular Biology and Biophysics University of Minnesota Minneapolis Minnesota USA

**Keywords:** liver transplantation, organ donor, RNA sequencing, senescence, transcriptomics

## Abstract

With an aging donor population, ischemic and inflammatory injury during organ procurement and preservation may be associated with senescence‐associated programs influencing graft biology. Although donor type‐specific injury patterns are known, their role in driving cellular senescence and downstream inflammatory responses remains unclear. We performed smart‐3SEQ bulk RNA‐sequencing on human liver tissue obtained from donation after brain death (DBD) and donation after circulatory death (DCD) donors. Senescence‐associated transcriptional signatures were assessed using the SenMayo gene panel and a liver‐specific senescence‐associated secretory phenotype panel. Immunohistochemistry assessed p16 and p21 protein expression. Findings were evaluated in NanoString‐based transcriptomic data from an external cohort. Sixty‐six livers (24 DBD and 42 DCD) were included. One thousand five hundredforty‐eight differentially expressed genes separated DBD and DCD grafts. Functional enrichment analysis showed distinct biological programs, with DBD grafts enriched for metabolic pathways, epithelial proliferation, and angiogenesis regulation, whereas DCD grafts showed enrichment of immune activation, chemokine signaling, and injury‐associated pathways. Senescence‐associated analyzes recapitulated this separation. Immunohistochemistry showed a higher proportion of p16‐positive nuclei in high‐risk DCD livers compared with DBD livers (*p* = 0.038), whereas no significant difference was observed for p21 (*p* = 0.687). However, tile‐level analysis showed significantly higher proportions of both p16‐ and p21‐positive nuclei in DCD livers (*p* < 0.001). External cohort analysis confirmed donor‐type separation across available transcripts and senescence‐associated gene panels. Donor type reflects distinct transcriptional states in human donor livers, with senescence‐associated profiles overlapping with donor type‐specific injury responses. Injury‐induced senescence‐associated signaling may influence graft function and recipient (patho)physiology, positioning it as a promising modifiable senolytic or senomorphic target in liver transplantation.

## Graphical abstract

Human donor livers exhibit distinct donor type‐specific transcriptional and senescence‐associated profiles, with donation after circulatory death grafts showing increased inflammatory signaling and senescence marker expression compared with donation after brain death grafts. Beyond the potential role as markers of graft quality, injury‐induced senescence‐associated pathways may influence graft function and recipient patho(physiology), representing promising targets in liver transplantation.

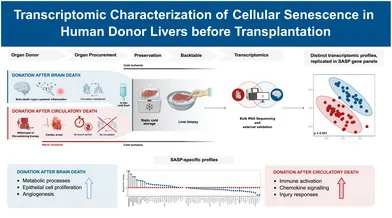

**Abbreviations**
DAB: 3,3′‐diaminobenzidine
DBD: donation after brain death
DCD: donation after circulatory death
PCA: principal component analysis
ROI: region of interest
SASP: senescence‐associated secretory phenotype
VST: variance‐stabilizing transformation

## 1 Introduction

Liver transplantation is the gold‐standard treatment for patients with end‐stage liver disease. However, its impact remains constrained by limited organ availability, which continues to drive waitlist mortality (European Association for the Study of the Liver 2024). To address organ scarcity, grafts from extended‐criteria donors, including livers from donation after circulatory death (DCD), are increasingly utilized alongside conventional donation after brain death (DBD) organs (Monbaliu et al. 2012). The donor type critically shapes the type of stresses to which a graft is exposed prior to transplantation. DBD is characterized by systemic inflammation and hemodynamic instability after brain death (Watts et al. 2013). DCD grafts are additionally exposed to a period of warm ischemia between withdrawal of life support and cold in situ flush during procurement (Figure 1A). This warm and prolonged ischemic phase is a major driver of ischemia–reperfusion injury and is strongly associated with post‐transplant complications, including primary non‐function and post‐transplant cholangiopathy (Esser et al. 2025). In particular, the development of post‐transplant cholangiopathy has been closely linked to ischemic injury and impaired biliary regenerative capacity (Esser et al. 2024; Ferreira‐Gonzalez et al. 2022).

**FIGURE 1:**
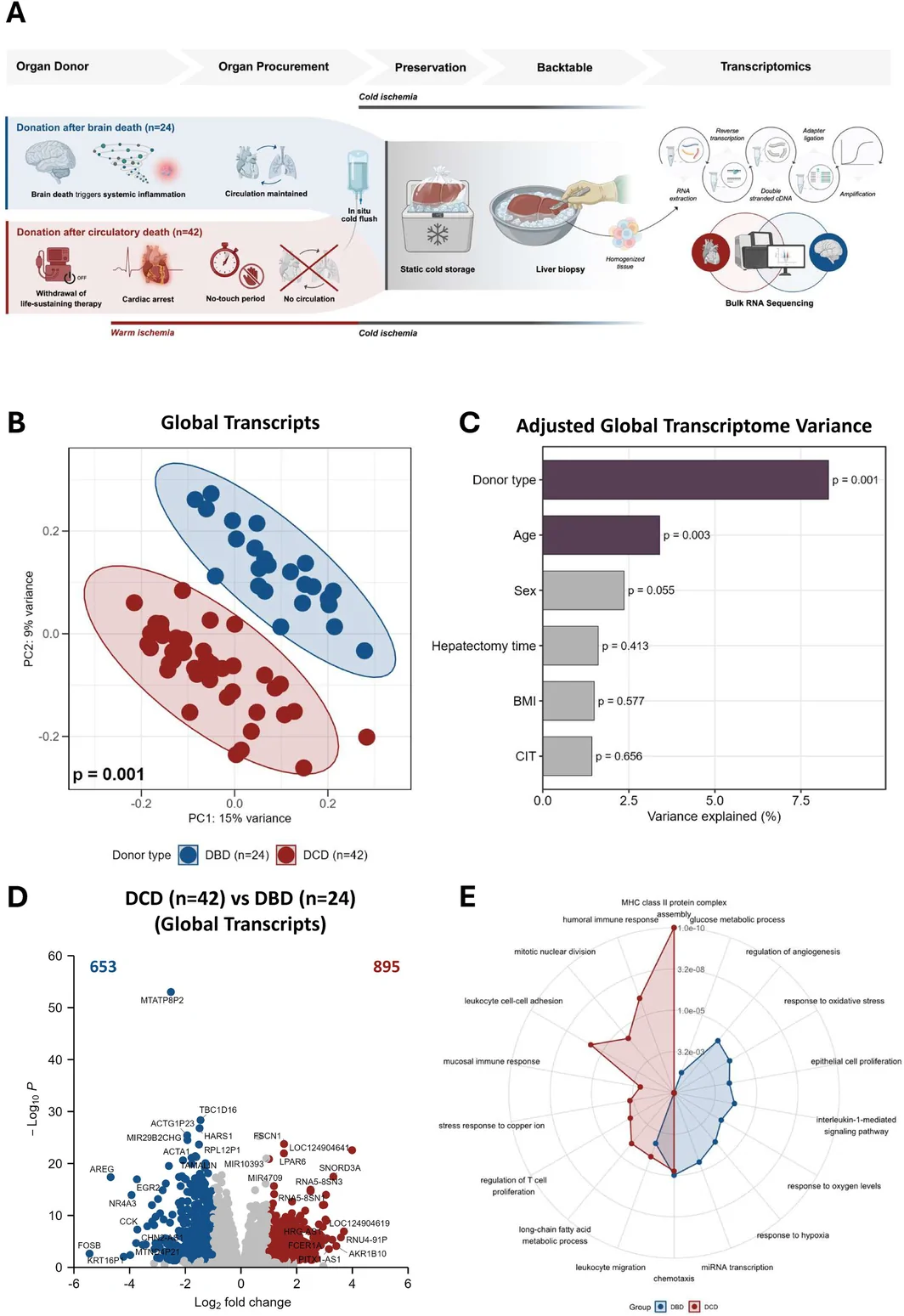
Characterization of the full transcriptome of DBD and DCD livers. (A) Depiction of donor‐type differences and the sample collection and analysis workflow. (B) PCA showing distinct clustering of DBD and DCD livers using the full identified transcriptome. (C) Permutational multivariate analysis of variance performed on variance‐stabilizing transformed expression data to quantify the contribution of donor type and other clinical variables to global transcriptional variation. (D) Volcano plot of all identified transcripts. Significant transcripts (*p* < 0.05, |log2FC > 1|) are highlighted in red for upregulated (enriched in DCD) and blue for downregulated (enriched in DBD). (E) Selected Gene Ontology pathways enriched in DCD and DBD livers. BMI, body mass index; cDNA, complementary deoxyribonucleic acid; CIT, cold ischemic time (min); DBD, donation after brain death; DCD, donation after circulatory death; PCA, principal component analysis; RNA, ribonucleic acid.

These donor‐type‐specific ischemic and inflammatory stresses may induce cellular damage responses in the graft, which may be reflected by changes in gene transcription and protein expression. One of the cellular pathways that may be altered in response to ischemia and inflammation is cellular senescence (Chen et al. 2022; Ferreira‐Gonzalez et al. 2022). Cellular senescence refers to a stable cell cycle arrest and stress response state that can be induced by diverse injurious stimuli, including ischemia–reperfusion‐associated stress, mitochondrial dysfunction, and reactive oxygen species‐mediated inflammatory signaling (Ajoolabady et al. 2025; Campisi and d'Adda di Fagagna 2007; Huang et al. 2022; Kayumov et al. 2025; Lopez‐Otin et al. 2013). This state is further characterized by the senescence‐associated secretory phenotype (SASP), which involves the secretion of factors that can disrupt tissue homeostasis and contribute to tissue and immune dysfunction (Ferreira‐Gonzalez et al. 2025; Wang et al. 2024). Senescence‐associated stress responses induced during organ procurement and preservation may therefore promote senescence‐associated programs and inflammation both in the graft and recipient (Chen et al. 2022; Ferreira‐Gonzalez et al. 2022; Iske et al. 2020; Kayumov et al. 2025). However, such responses might not be uniformly detrimental, and their biological effects may depend on timing and persistence. While persistent or established cellular senescence, particularly beyond the acute injury phase, is generally associated with tissue dysfunction, senescence‐associated responses can also arise rapidly in response to acute tissue injury (Nehme et al. 2024; Valdivieso et al. 2026). In this context, transient senescence‐associated programs may represent an adaptive damage response that limits cell death and contributes to tissue regeneration and repair (Nehme et al. 2024).

Given that the donor type determines the nature of ischemic and inflammatory stress, we hypothesized that DBD and DCD grafts are characterized by distinct senescence‐associated and inflammatory transcriptional signatures. However, it is unknown to what extent the donor type‐specific injury is reflected in the biological state of livers prior to transplantation. To address this gap, we performed transcriptomic profiling of human donor liver tissue from DBD and DCD donors following static cold storage (SCS) to determine whether donor‐specific injury translates into distinct global and SASP‐associated transcriptional signatures that originate during donor death and persist through procurement and preservation.

## 2 Methods

### 2.1 Study Cohort

All donor livers were procured by one of the regional multi‐organ procurement teams in the Netherlands, using a previously described standardized procedure (van Rijn et al. 2017). A mandatory 5‐min no‐touch period after circulatory arrest was observed for all DCD donors. Following procurement, livers were transported to our center using SCS. Liver tissue biopsies were collected upon arrival at our center during backtable preparation. A depiction of the full workflow is shown in Figure 1A. Donor and procurement characteristics, including donor age, donor sex, cold ischemia time, hepatectomy time, and (for DCD) warm ischemia time, were recorded for subsequent comparisons and transcriptomic analyzes.

This study was conducted in accordance with the Declaration of Helsinki and the Declaration of Istanbul. All donor samples and associated clinical data were prospectively collected as part of the TransplantLines cohort study and biobank (University Medical Center Groningen Medical Ethics Committee approval 2014/077, ClinicalTrials.gov identifier NCT03272841) (Eisenga et al. 2018; Posthumus et al. 2025). Because only donor data and biological samples were analyzed, the study was exempt from additional institutional review.

### 2.2 RNA‐Sequencing

RNA was isolated using the Qiagen RNeasy Lipid Tissue Mini Kit (Qiagen, Hilden, Germany) according to the manufacturer's protocol. RNA‐sequencing libraries were constructed according to the Smart‐3SEQ protocol using 100 ng of total RNA per sample as input (Foley et al. 2019). Libraries were pooled and sequenced on an Illumina NextSeq 500 instrument at iPsomics (Groningen, the Netherlands). Reads were trimmed using TrimGalore v0.6.7, deduplicated with SeqKit v2.4.0, and mapped to the human genome assembly GRCh38.p14 using HISAT2 v2.2.1. Gene counts were generated using featureCounts v2.0.1 with NCBI RefSeq gene annotations.

### 2.3 RNA‐Sequencing Data Analysis

Count data were normalized using the median‐of‐ratios method in DESeq2 in R (v4.5.2). Differential gene expression analysis was performed using DESeq2, with donor type as the primary variable and sex and age included as a covariates (design: ~ Donor.type + Sex + Age). Multiple testing correction was performed using the Benjamini‐Hochberg procedure, and genes with adjusted *p* < 0.05 and |log2FC > 1| were considered differentially expressed for downstream directional analyzes.

Variance‐stabilizing transformation (VST) expression values were generated in DESeq2 and used for unsupervised analyzes and visualization. Principal component analysis (PCA) was performed on VST‐transformed expression values. Where appropriate for visualization, genes were prioritized based on variability across samples. Differential expression results were displayed using volcano plots based on adjusted *p* values and log2FC. Functional interpretation was performed using Gene Ontology enrichment analysis with *p* values adjusted for multiple‐testing correction using the Benjamini‐Hochberg procedure to control the false discovery rate.

Permutational multivariate analysis of variance (PERMANOVA) was performed on VST‐transformed expression data to assess the contribution of donor type and other clinical variables to global transcriptional variation (adonis2 function, vegan R package, Euclidean distance, 999 permutations). Donor type, donor age, donor sex, BMI, cold ischemia time, and hepatectomy time were included in a single multivariable model, and the contribution of each variable was assessed conditional on all other variables.

To assess senescence‐associated transcriptional signatures, we first used the SenMayo gene set as a transcriptional framework. This gene panel is derived from multiple senescence studies for systematic assessment of senescence‐associated pathways across tissues and biological contexts and comprises 125 genes, including a subset associated with the SASP (Saul et al. 2022). Because the SenMayo panel is not liver‐specific, we additionally assembled a set of 51 liver‐specific SASP‐associated genes based on previously published literature (Du et al. 2026; Sanfeliu‐Redondo et al. 2024).

For both the SenMayo and the liver‐specific SASP‐associated panel, we identified transcripts most strongly contributing to donor‐type separation by calculating the mean VST expression for DBD and DCD grafts. Genes were ranked by the absolute between‐group difference and separated into DBD‐higher and DCD‐higher groups for enrichment analyzes.

### 2.4 Immunohistochemistry

Formalin‐fixed paraffin‐embedded tissue sections were cut at 4 μm and stained for p16 (Rabbit anti‐p16 Sc‐1207, Santa Cruz, dilution 1:150) and p21 (Rabbit anti‐p21 Sc‐471, Santa Cruz, dilution 1:400). Immunohistochemical staining was performed using 3,3′‐diaminobenzidine (DAB) with hematoxylin counterstaining. Images were scanned using a Hamamatsu slide scanner (Aperio GT 450 DX, Leica Biosystems, Germany). Whole‐slide images were analyzed in QuPath (v0.6.0) using the brightfield (H‐DAB) image type. Image analysis was performed blinded to donor type. Stain vectors were estimated per slide to enable hematoxylin/DAB separation and region of interests (ROIs) were manually annotated to include relevant tissue while excluding artifacts. For assessment, ROIs were further subdivided into square annotation tiles (1000 μm) trimmed to the ROI boundary (Supplementary Figure 1A,B). Nuclei were detected using the optical density sum image for segmentation, and marker positivity was quantified using the mean nuclear DAB optical density. Positively stained nuclei were manually identified and single optical density thresholds of ≥ 0.42 for p16 and ≥ 0.36 for p21 were selected and applied to all individual sections for p16 and p21 staining, respectively. For each full ROI and square annotation tile, the proportion of positively stained nuclei was calculated over the sum of total identified nuclei. As a complementary analysis, transcriptional profiles of *CDKN1A* (p21^CIP1^) and *CDKN2A* (p16^INK4a^/p19^ARF^) were analyzed to assess gene expression levels corresponding to the staining patterns.

### 2.5 External Validation

Publicly available targeted transcriptomic data from an independent cohort were analyzed to determine whether the transcriptional patterns identified in our cohort were also observed externally. In this cohort, gene expression was measured using the NanoString nCounter PanCancer Immune Profiling panel in human donor liver tissue from 18 DCD and 29 DBD grafts obtained after procurement (Keslar et al. 2025). PCA and differential gene expression analyzes were performed using all available transcripts, and were repeated after restriction to the SenMayo gene set and the curated liver‐specific SASP‐associated gene panel.

### 2.6 Statistical Analysis

Descriptive statistics were used to summarize donor characteristics. Continuous variables are reported as median and interquartile range (IQR), and categorical variables as numbers (percentages). Differences between DBD and DCD donors were assessed using independent samples *t*‐tests for continuous variables and chi‐square tests for categorical variables. Fisher's exact test was used when expected cell counts were < 5. Differences in the proportion of positively stained nuclei between DBD and DCD were assessed using the Mann–Whitney *U*‐test. All statistical tests were two‐tailed, and *p* values < 0.05 were considered statistically significant. Statistical analyzes were performed using R (v4.5.2) and Prism GraphPad (v9). All authors had access to the study data and had reviewed and approved the final manuscript.

## 3 Results

### 3.1 Study Cohort

In total, 66 human donor livers procured between May 2019 and July 2022 were included in this study, comprising 24 grafts from DBD and 42 grafts from DCD donors. Donor and preservation characteristics are summarized in Table 1. Median donor age was 55 years (IQR 48–62) in the DBD group and 67 years (IQR 60–69) in the DCD group (*p* = 0.001). Median static cold ischemia time was 272 min (IQR 232–288) for DBD and 256 min (IQR 207–288) for DCD grafts (*p* = 0.121). The median functional donor warm ischemia time in DCD was 29 min (IQR 25–34). Median hepatectomy time was comparable between DBD and DCD grafts with 30 min (IQR 26–35) and 39 min (IQR 32–42), respectively (*p* = 0.260). The median donor risk index was 1.8 (IQR 1.4–2.0) in DBD and 2.8 (IQR 2.5–3.1) in DCD livers (*p* < 0.001).

**TABLE 1:** Donor characteristics.

	DBD (*n* = 24)	DCD (*n* = 42)	*p*
Sex			
Female	11 (45.8%)	15 (35.7%)	0.444
Male	13 (54.2%)	27 (64.3%)	0.444
Age (years)	54.5 (47.8–61.5)	66.5 (60.2–69.0)	0.001
BMI (kg/m^2^)	25.9 (24.0–28.2)	26.2 (23.8–27.8)	0.954
Cause of death category			
Anoxia	4 (16.7%)	13 (31.0%)	0.251
CVA	16 (66.7%)	19 (42.5%)	0.126
Other	2 (8.3%)	5 (11.9%)	1.000
Trauma	2 (8.3%)	5 (11.9%)	1.000
Hepatectomy time (min)	30.0 (26.0–35.0)	39.0 (32.2–42.0)	0.260
AST (U/L)	43.5 (25.8–104.5)	34.5 (24.2–113.2)	0.158
ALT (U/L)	35.0 (20.0–105.0)	27.5 (18.0–74.0)	0.181
Time from withdrawal of life support to circulatory arrest (min)	—	15.0 (11.2–21.8)	
Functional donor warm ischemia time (min)	—	29.0 (25.0–34.0)	
Static cold ischemia time (min)	272.0 (232.0–288.0)	255.5 (207.0–288.0)	0.121
DRI	1.8 (1.4–2.0)	2.8 (2.5–3.1)	< 0.001

Abbreviations: ALT, alanine aminotransferase; AST, aspartate aminotransferase; BMI, body mass index; CVA, cerebrovascular accident; DCD, donation after brain death; DCD, donation after circulatory death; DRI, donor risk index.

### 3.2 Global Transcriptomic Analysis Shows Distinct Donor Type‐Specific Signatures

Bulk RNA‐sequencing across all donor liver samples identified a total of 59,251 transcripts. Unsupervised hierarchical clustering based on the full transcriptome demonstrated clear separation by donor type (PC1: 15% variance, PC2: 9% variance; PERMANOVA: *p* = 0.001; Figure 1B). PERMANOVA showed that the donor type was the primary determinant of global transcriptional variation and explained the largest proportion of variance (*p* = 0.001). While age was associated with global transcriptional variation, other clinical variables, including sex, BMI, cold ischemia time, and hepatectomy time, were not significantly associated with global gene expression patterns. Overall, donor type showed the strongest association with transcriptomic variability in liver tissue following SCS (Figure 1C).

Differential gene expression analysis identified broad transcriptional differences between donor types, with 1548 significantly differentially expressed genes (*p* < 0.05 and |log2FC > 1|; Figure 1D). DBD livers showed higher expression of immediate‐early and response‐associated genes, including *AREG* and *FOSB*. In contrast, DCD livers showed increased expression of injury and stress‐related genes, including *FSCN1* and *AKR1B10*.

Functional enrichment of donor type‐associated transcripts demonstrated a marked difference in biological profiles between donor types. DBD livers showed enrichment of pathways associated with epithelial proliferation (GO:0050673), responses to hypoxia and oxidative stress (GO:0001666 and GO:0006979), angiogenesis regulation (GO:0045765), as well as glucose metabolism (GO:0006006) (Figure 1E). DCD livers showed significant enrichment of immune response and stress‐related pathways, including leucocyte cell–cell adhesion and migration (GO:0007159 and GO:0050900), humoral immune response (GO:0006959), and MHC Class II protein complex assembly (GO:0002399). Taken together, global transcriptomic profiling demonstrated clear separation between DBD and DCD livers, with donor type explaining a substantial proportion of the transcriptomic variability.

### 3.3 Senescence‐Associated Transcriptional Profiles Differ Between Donor Types

To investigate senescence‐associated transcriptional profiles, analyzes were performed within the SenMayo gene set as a framework for senescence‐ and SASP‐associated genes, while recognizing that these transcripts simultaneously partly reflect broader inflammatory and injury‐related responses (Saul et al. 2022). PCA restricted to the SenMayo transcripts separated donor types (PC1: 23% variance, PC2: 12% variance; PERMANOVA: *p* = 0.001; Figure 2A). Within this panel, a subset of genes captured the donor‐type‐associated transcriptional variation. This separation was driven by a subset of 24 significantly differentially expressed genes among the 125 genes included in the SenMayo gene set. In DBD livers, enriched transcripts included *AREG* and *SERPINE1*. In DCD livers, *CXCL12* and additional chemokine‐related or endothelial/stromal‐associated genes, including *ESM1*, *CCL24*, *CCL8*, *CXCL10*, as well as *SERPINE2*, were enriched (Figure 2B).

**FIGURE 2:**
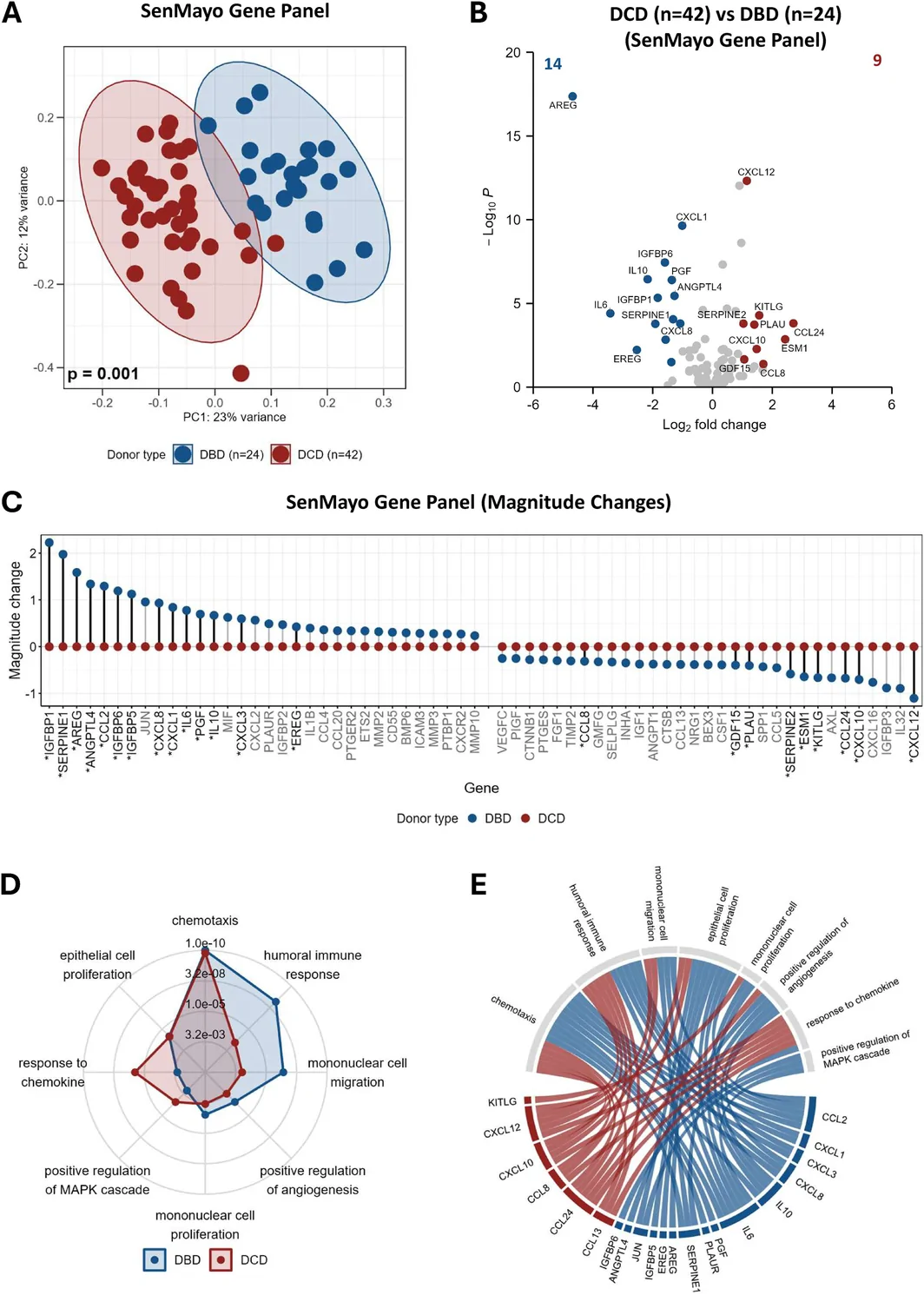
Characterization of SenMayo gene panel in DBD and DCD livers. (A) PCA depicting distinct clustering between DBD and DCD livers when restricted to the SenMayo gene panel. (B) Volcano plot of identified SenMayo gene set transcripts. Significant transcripts (*p* < 0.05, |log2FC > 1|) are highlighted in red for upregulated (enriched in DCD) and blue for downregulated (enriched in DBD). (C) Range plot depicting the magnitude differences of SenMayo gene panel transcripts. For each gene, mean variance‐stabilized expression was calculated in DBD and DCD samples. The DCD value is fixed at 0, and the DBD value is plotted as the signed difference (DBD minus DCD). Genes that were found to be significantly differentially expressed in the volcano plot in (B) are highlighted in bold and marked with an asterisk. (D) Selected Gene Ontology pathways for the SenMayo subset are shown as a radar plot of enrichment significance (−log10(adjusted *p*)), with separate traces for DBD (blue) and DCD (red). (E) Circos plot mapping the selected SenMayo Gene Ontology pathways and their contributing genes, restricted to genes found to be significantly differentially expressed in the volcano plot in (B). Genes are colored based on direction of differential expression. DBD, donation after brain death; DCD, donation after circulatory death; PCA, principal component analysis.

To identify the transcripts most strongly contributing to this separation, genes were evaluated according to the magnitude of their donor‐associated expression differences. For this, a magnitude metric reflecting the size and direction of the expression was calculated from the mean VST expression values. SenMayo genes were ranked by absolute magnitude of expression differences between DBD and DCD livers (Figure 2C). This revealed a bidirectional distribution, with distinct subsets of genes showing a higher expression in each donor type. Several significantly differentially expressed genes (Figure 2B) were among the strongest contributors to this separation. Among these, *IGFBP1*, *SERPINE1*, *AREG*, *ANGPTL4*, *CCL2*, and *IGFBP6* showed a higher expression in DBD livers, whereas *CXCL12*, *CXCL10*, *CCL24*, *KITLG*, *ESM1*, and *SERPINE2* showed a higher expression in DCD livers (Figure 2C). This shows that the most pronounced donor‐associated shifts within the SenMayo subset were concentrated in a limited set of significantly altered transcripts.

Pathway enrichment analysis of these SenMayo genes showed substantial pathway overlap despite different gene expression levels between donor types. DBD‐associated SenMayo signals were enriched for pathways related to immune‐cell migration, including humoral immune response (GO:0006959) and mononuclear cell migration (GO:0071674). DCD‐associated SenMayo signals were relatively stronger for chemokine signaling (GO:1990868) and positive regulation of the MAPK cascade (GO:0043410). Shared enrichment was observed for pathways related to chemotaxis (GO:0006935), epithelial proliferation (GO:0050673), mononuclear cell proliferation (GO:0032943), and positive regulation of angiogenesis (GO:0045766) (Figure 2D).

Mapping of individual genes contributing to the identified pathways showed that differentially expressed SenMayo genes in DBD were predominantly canonical inflammatory chemokines and cytokines (e.g., *CCL2*, *CXCL1/3/8*, *IL6*, *IL10*), as well as *AREG*, *SERPINE1*, *IGFBP*, and *ANGPTL4*. DCD‐associated SenMayo genes included chemokines, such as *CXCL10/12*, *CCL8/13/24*, as well as *KITLG* (Figure 2E). A comprehensive overview of all SenMayo genes associated with pathways in DBD and DCD livers is presented in Supplementary Figure 2.

Given that the SenMayo gene set lacks liver‐specificity, we additionally derived a liver‐relevant SASP‐associated injury panel based on previously published literature on cellular senescence in liver disease. This panel included 51 genes representing senescence‐associated secretory and inflammatory programs previously described in the context of hepatic injury (Supplementary Table 1) (Du et al. 2026; Sanfeliu‐Redondo et al. 2024). This panel showed a comparable separation between DBD and DCD (PC1: 24% variance, PC2: 16% variance, PERMANOVA: *p* = 0.001; Figure 3A). The inflammatory‐driven response in DBD and the more stress‐ and damage‐driven response in DCD were similarly reflected within this gene panel (Figure 3B). DCD showed stronger enrichment for pathways related to epithelial cell proliferation (GO:0050673) and regulation of MAPK signaling (GO:0043410), whereas in DBD stronger enrichment of pathways related to mononuclear cell proliferation (GO:0032943) was found (Figure 3C). Although the strongest expression differences were shifted toward DBD, most differentially expressed genes were found in DCD (Figure 3D). The complete set of differentially expressed genes between DCD and DBD is shown in Supplementary Figure 3.

**FIGURE 3:**
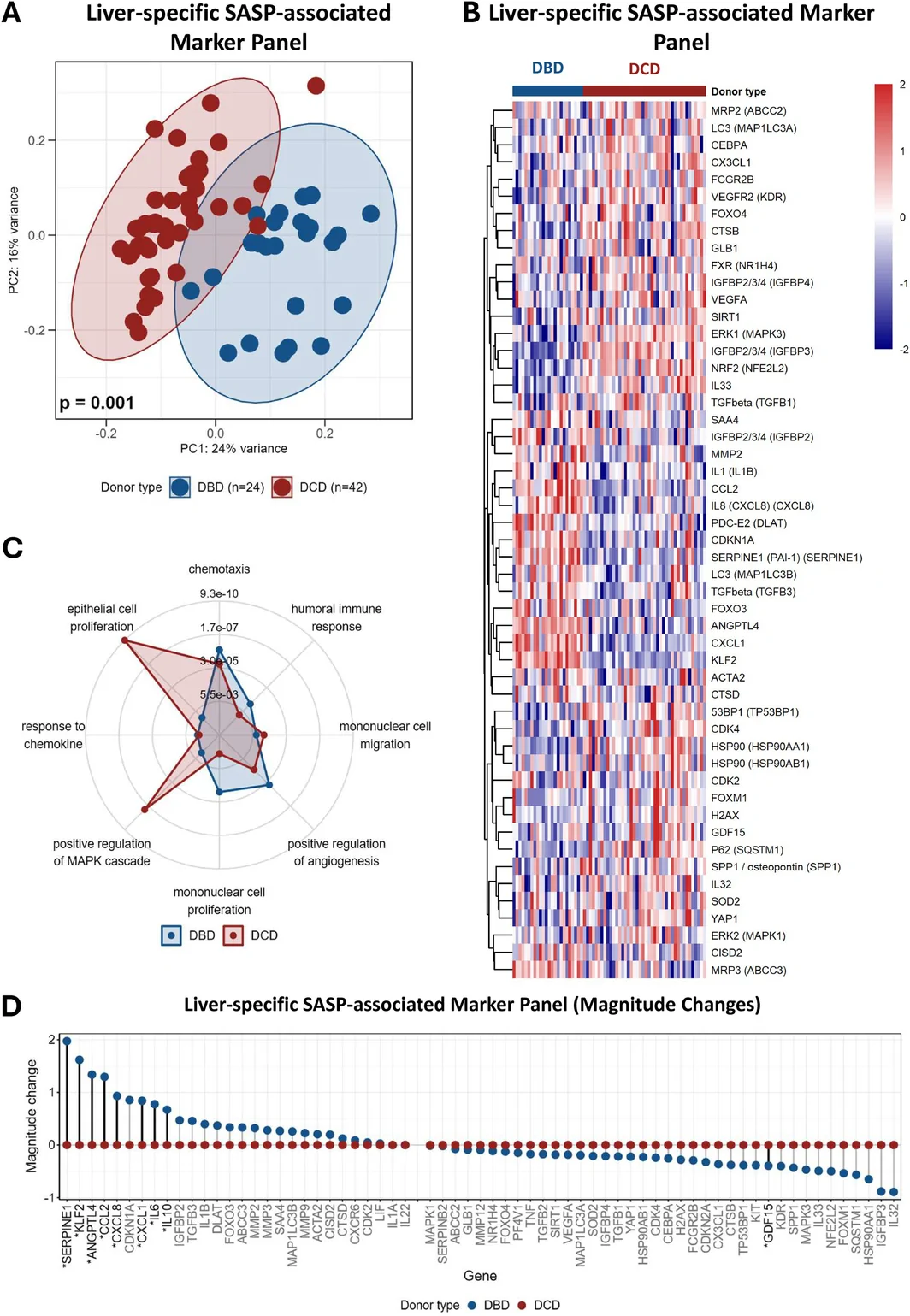
Liver‐relevant SASP‐associated panel in DBD and DCD livers. (A) PCA clustering of DBD and DCD livers when restricted to the liver‐specific SASP‐associated panel. (B) Heatmap showing the relative expression of the individual markers across DBD and DCD donors. Red indicates enrichment in DCD and blue enrichment in DBD. (C) Selected Gene Ontology pathways for the liver‐relevant SASP‐associated panel are shown as a radar plot of enrichment significance (−log10(adjusted *p*)), with separate traces for DBD (blue) and DCD (red). (D) Range plot depicting the magnitude difference of liver‐relevant SASP‐associated gene set transcripts. For each gene, mean variance‐stabilized expression was calculated in DBD and DCD samples. The DCD value is fixed at 0, and the DBD value is plotted as the signed difference (DBD minus DCD). Genes that were found to be significantly differentially expressed are highlighted in bold and marked with an asterisk. DBD, donation after brain death; DCD, donation after circulatory death; PCA, principal component analysis; SASP, senescence‐associated secretory phenotype.

### 3.4 DCD Livers Show Increased Immunohistochemical Expression of Senescence‐Associated Stress Markers p16 and p21

To evaluate senescence‐associated changes at the tissue level, random subsets of DBD and DCD livers were analyzed for p16 and p21 by immunohistochemical staining. For p16, the analysis included 9 DBD and 11 DCD livers and for p21 it included 8 DBD and 13 DCD livers. Representative images showed limited nuclear positivity in both donor types, with staining largely confined to periportal and portal regions (Figure 4A,B; Supplementary Figure 1A,B). Quantification of p16‐ and p21‐positive stained nuclei at the full ROI level showed a significantly higher proportion of p16‐positive nuclei in high‐risk DCD livers compared with DBD livers (*p* = 0.038; Figure 4C), whereas no significant difference was observed for p21‐positive nuclei between donor types (*p* = 0.687; Figure 4D). To further characterize the spatial heterogeneity of p16‐ and p21‐positive nuclei, the annotated ROIs were subdivided into smaller tiles (median tiles per slide: 15, IQR 12–17 for p16; median tiles per slide: 17, IQR 13–18 for p21). At the tile level, high‐risk DCD showed a significantly higher proportion of both p16‐ and p21‐positive nuclei compared with DBD livers (Supplementary Figure 1C,D).

**FIGURE 4:**
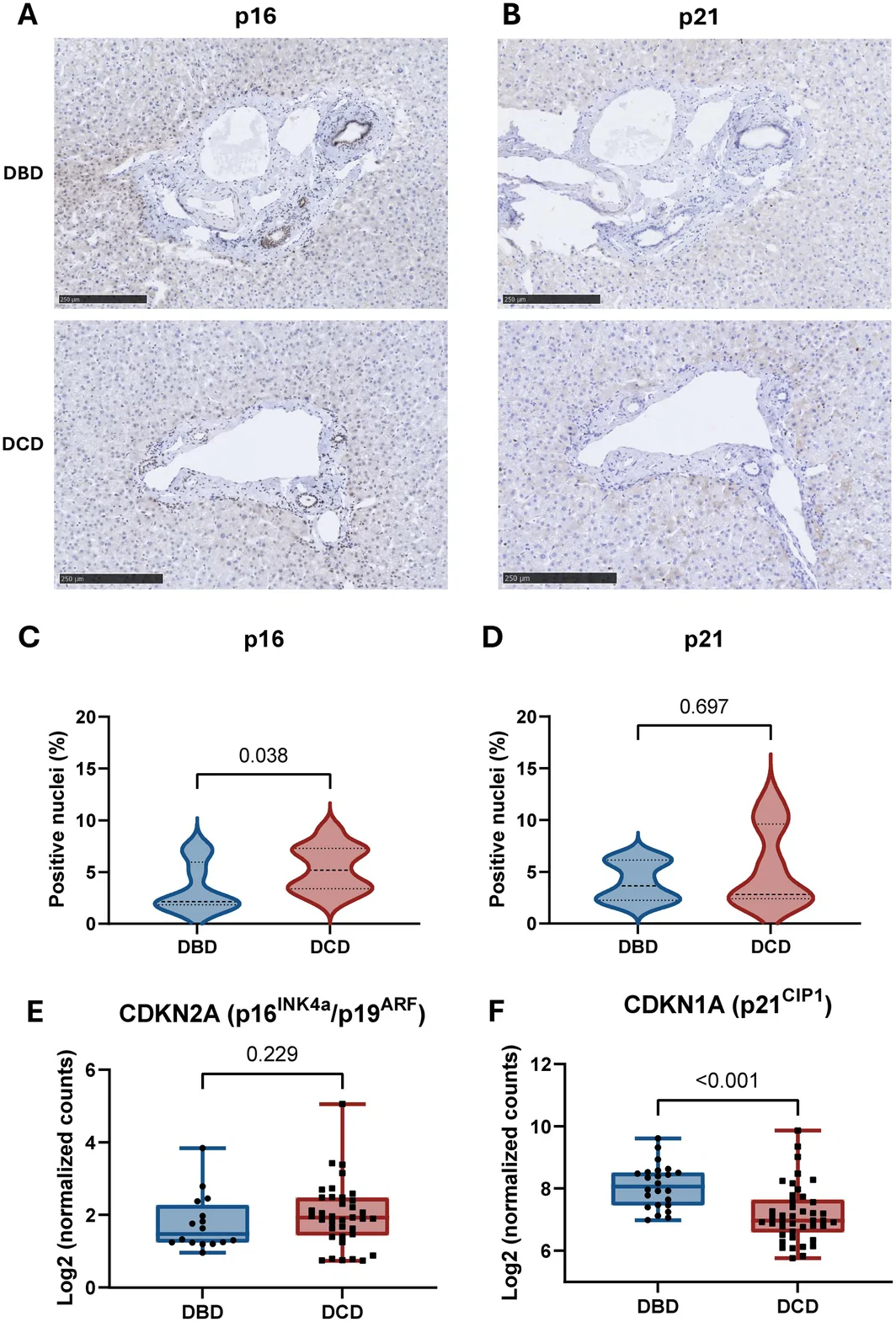
Immunohistochemical analysis of p16 and p21 in liver tissue. (A, B) Representative immunohistochemistry images of (A) p16 and (B) p21 staining in DBD and DCD liver tissue sections. Scale bars represent 250 μm. (C, D) Quantification of (C) p16‐positive and (D) p21‐positive stained nuclei in DBD and DCD. (E, F) Expression of (E) *CDKN2A* (p16^INK4a^/p19^ARF^) and (F) *CDKN1A* (p21^CIP1^) in DBD and DCD, with a higher expression of *CDKN2A* (p16^INK4a^/p19^ARF^) in DCD grafts (*p* = 0.229) and an upregulation of *CDKN1A* (p21^CIP1^) in DBD grafts (*p* < 0.001). DBD; donation after brain death, DCD; donation after circulatory death.

To complement the immunohistochemical findings, we assessed these canonical senescence markers in bulk RNA‐sequencing data. While *CDKN2A* (p16^INK4a^/p19^ARF^) showed a trend toward higher expression in DCD grafts (*p* = 0.229), *CDKN1A* (p21^CIP1^) expression was significantly upregulated in DBD grafts (*p* < 0.001) (Figure 4E,F).

### 3.5 External Cohort Analysis Shows Donor‐Type Separation Across All Available and Senescence‐Associated Transcripts

A total of 711 transcripts were available in the external NanoString dataset (Keslar et al. 2025). The targeted NanoString panel covered only a subset of genes from the predefined panels, including 57 of the 125 SenMayo genes (Supplementary Table 2) and 15 of the 51 genes from the liver‐specific SASP‐associated panel (Supplementary Table 3).

PCA based on all available transcripts showed a clear separation between DBD and DCD grafts (PC1: 15% variance, PC2: 15% variance; PERMANOVA: *p* = 0.001; Figure 5A). Similar donor‐type separation was observed when restricting to the transcripts overlapping with the SenMayo gene set (PC1: 33% variance, PC2: 16% variance; PERMANOVA: *p* = 0.003; Figure 5B) and the liver‐specific SASP‐associated panel (PC1: 43% variance, PC2: 18% variance; PERMANOVA: *p* = 0.002; Figure 5C).

**FIGURE 5:**
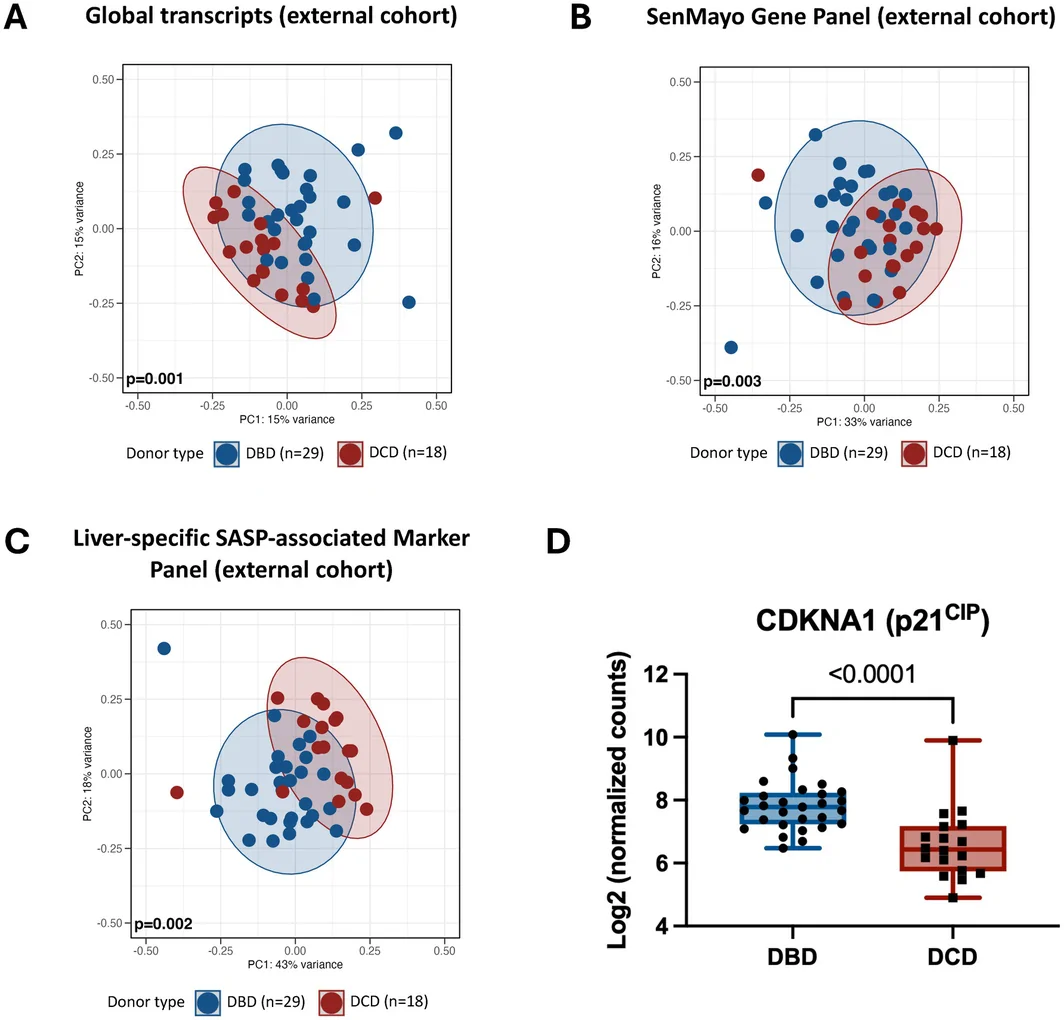
External cohort analysis of all available transcripts and senescence‐associated gene panels. (A) PCA clustering of DBD and DCD livers using all available gene transcripts in the external dataset. The analysis was repeated after restricting to transcripts overlapping with (B) the SenMayo gene panel, and (C) the curated liver‐specific SASP‐associated gene panel. (D) *CDKN1A* (p21^CIP1^) expression was higher in DBD compared to DCD grafts (*p* < 0.001). DBD, donation after brain death; DCD, donation after circulatory death; PCA, principal component analysis; SASP, senescence‐associated secretory phenotype.

Differential gene expression analysis identified 11 significantly differentially expressed genes between DBD and DCD livers (*p* < 0.05 and |log2FC > 1|). Most differentially expressed genes showed higher expression in DBD grafts, including *FOS*, *EGR1*, *THBS1*, *DUSP6*, *CDKN1A*, *IL1R1*, *CXCR4*, *CCL2*, and *IL8*, whereas *TNFSF14* showed higher expression in DCD grafts (Supplementary Figure 4). We further assessed *CDKN1A* (p21^CIP1^) expression in the external validation cohort. In line with the findings within our cohort, *CDKN1A* (p21^CIP1^) was significantly higher in DBD than in DCD grafts (*p* < 0.001; Figure 5D).

## 4 Discussion

Donor type is a major factor associated with distinct transcriptional profiles of donor livers prior to transplantation. Global transcriptomic profiling showed clear separation between DBD and DCD grafts, with donor type explaining more transcriptional variability than conventional donor characteristics such as age, sex, and BMI, or preservation‐related characteristics, including ischemia time and hepatectomy time. Although DCD donors were older in this cohort (Table 1) and age contributed to transcriptional variability, donor type remained the strongest contributor after accounting for these factors. This indicates that donor type‐specific injury patterns predominantly shaped the observed differences. Together, this shows that the donor type substantially contributes to the transcriptomic variability following cold storage, with procurement‐related injury substantially driving the heterogeneity between graft types (Figure 1B).

This donor type‐driven divergence was further reflected in functional pathway enrichment, where DBD and DCD livers showed donor type‐specific separation. DBD livers showed enrichment of pathways related to epithelial proliferation, metabolic processes, and responses to oxidative stress, whereas DCD livers were characterized by a stronger enrichment of immune activation and leukocyte‐related pathways. Mechanistically, these differences reflect the distinct injury mechanisms preceding organ procurement. In DBD, brain death induces systemic inflammatory and hemodynamic disturbances (Watts et al. 2013), whereas in DCD, graft biology is shaped by the period of warm ischemia and hypoxia that occurs following circulatory arrest.

A similar separation of donor types was observed when the analysis was restricted to SASP‐associated gene sets, showing that only a limited number of differentially expressed genes drive the same donor type‐specific separation. Specifically, only 24 of the 125 SenMayo genes were significantly differentially expressed, yet this subset was sufficient to separate DBD and DCD grafts. The additional liver‐related SASP‐associated injury panel, comprising 51 genes, comparably separated the donor types. Together, these findings indicate that donor‐type‐specific injury patterns induce coordinated senescence‐associated transcriptional programs rather than broad, uniform transcriptional changes. These transcriptional changes may have functional consequences beyond the cells in which they originate, as SASP components, including cytokines, chemokines, growth factors, and proteases, can affect surrounding cells and tissues (Nehme et al. 2024). Donor‐related injury may therefore not only lead to a fundamentally different state of the graft, but also contribute to the biological variation between DBD and DCD livers through senescence‐associated processes. Importantly, donor type‐specific senescence‐associated transcriptional signatures were also observed in an independent cohort, although validation was limited to the subset of genes represented on the targeted NanoString panel.

Overall, the biological relevance of these findings may extend beyond the graft itself, as senescence‐associated signals can affect neighboring and distant cells through the paracrine and systemic transmission of SASP factors (Ferreira‐Gonzalez et al. 2025; Kiourtis et al. 2024). This is further supported by evidence that hepatocellular senescence can induce multi‐organ dysfunction, illustrating the potential consequences of senescence‐associated factors acting beyond the cells in which they originate (Kiourtis et al. 2024). This concept may be particularly relevant in the context of organ transplantation, where senescent cells and SASP factors accumulate in the donor organ. Preclinical studies have further demonstrated that senescence can propagate within recipient tissue through SASP factors (Iske et al. 2021, 2024), suggesting that senescence may not only contribute to graft dysfunction but also shape recipient (patho)physiology following transplantation. Consistent with this, transplantation of older donor organs into younger recipients has been associated with inferior graft survival, potentially reflecting the transfer of senescent cell burden and its downstream effects (Shirini et al. 2026). However, the biological impact of senescence strongly depends on timing and tissue context. Recent work has shown that rapidly induced senescence is essential for efficient tissue repair, and that shifting acute oxidative injury from cell death toward transient senescence promotes tissue repair (Nehme et al. 2024). At the same time, hypothermic oxygenated machine perfusion, which mitigates ischemia–reperfusion injury, has been associated with improved graft survival of older donor organs (Eden et al. 2025). This suggests that interventions modulating oxidative stress and senescence dynamics may influence whether senescence acts as a repair response or as a chronic driver of tissue dysfunction. However, whether the donor type‐specific transcriptional profiles identified in the present study are associated with post‐transplant outcomes remains to be determined.

Accordingly, cellular senescence is increasingly recognized as a therapeutic target, with both senolytics and senomorphics emerging as potential gerotherapeutic strategies (Lau et al. 2019). While senolytics aim to selectively eliminate senescent cells, senomorphics attenuate the SASP and related injury‐associated pathways without eliminating senescent cells (Mansfield et al. 2024). Both the pharmacological clearance and genetic targeting of senescent cells have been shown to restore regenerative capacity and improve transplant outcomes and graft survival in experimental models (Braun et al. 2012; Esser et al. 2024; Ferreira‐Gonzalez et al. 2022; Iske et al. 2020). Specifically, experimental studies have demonstrated that senescence of biliary cells arising during organ donation contributes to the loss of biliary regenerative capacity during subsequent cold storage (Esser et al. 2024; Ferreira‐Gonzalez et al. 2022). To this regard, in the setting of liver transplantation, ex situ machine perfusion provides a platform to deliver such therapies directly to the graft prior to transplantation. Machine perfusion may allow senolytic and senomorphic strategies to be applied in a controlled manner, potentially reducing donor‐derived senescent cell burden while preserving transient senescent programs that support tissue repair through modulation of senescence‐associated pathways (Lascaris et al. 2022).

Current recommendations for assessing cellular senescence suggest a multi‐layered approach that first evaluates core cell‐cycle arrest markers, in particular p16 and p21, followed by verification using additional auxiliary markers and SASP‐associated factors (Ogrodnik et al. 2024). In line with this, we assessed senescence‐associated changes using p16 and p21 immunohistochemistry as indicators of senescence‐associated cell‐cycle regulation at a protein level. We found a significantly higher proportion of both p16‐ and p21‐positive nuclei in DCD grafts (Supplementary Figure 1C,D).

This difference at the protein level was not fully reflected by the transcriptomic data for *CDKN1A* (p21^CIP1^) and *CDKN2A* (p16^INK4a^/p19^ARF^) with a significantly higher expression in DBD and a trend toward a higher expression in DCD, respectively (Figure 4E,F). This discrepancy may be explained by several biological factors that influence the assessment of senescence. Bulk RNA‐sequencing captures averaged gene expression across the entire sample, limiting its ability to resolve the cellular origin of senescence‐associated signatures. As a result, cell‐type‐specific accumulation of senescence‐associated cells may be diluted, particularly because these signatures may arise from hepatocytes, cholangiocytes, endothelial cells, immune cells, or fibroblasts with distinct transcriptional profiles (Karpova et al. 2026). In contrast, immunohistochemistry provides spatial information and can detect localized accumulation of senescence markers within tissue compartments. However, transcript and protein levels may be uncoupled. p21 protein was found to accumulate rapidly in injured tissue through post‐transcriptional mechanisms and translation of pre‐existing *CDKN1A* (p21^CIP1^) transcripts, without an increase in messenger RNA abundance (Valdivieso et al. 2026). Furthermore, *CDKN2A* (p16^INK4a^/p19^ARF^) reflects multiple different cell populations, including inflammatory and immune cells (Valdivieso et al. 2026). Therefore, the expression of *CDKN2A* (p16^INK4a^/p19^ARF^) may not correspond directly to p16 protein.

The discrepancy between transcriptomic and protein level findings further highlights the relevance of tissue compartmentalization. Recent spatial and single‐cell analyzes have demonstrated that senescent cells in the human liver form spatially organized clusters that are influenced by cell–cell interactions and are preferentially localized to periportal regions (Kayumov et al. 2025). Consistent with these findings, immunohistochemical staining demonstrated that p16 and p21 nuclei positivity was predominantly localized to portal and periportal regions. Such compartmentalized responses may be particularly relevant for the development of post‐transplant cholangiopathy, which represents a major complication after transplantation of DCD grafts. DNA damage responses and SASP‐associated signaling in cholangiocytes may contribute to biliary injury by impairing the regenerative capacity of the biliary epithelium (Ferreira‐Gonzalez et al. 2022).

However, because neither bulk RNA‐sequencing nor p16 and p21 immunohistochemistry can definitively identify senescent cells on their own, our findings should be interpreted as evidence of donor‐type specific senescence‐associated transcriptional and protein changes rather than direct quantification of senescent‐cell burden. Given the association between aging and cellular senescence, the higher age of DCD donors represents a potential residual confounding factor. Nevertheless, after accounting for donor age, donor type remained the strongest contributor to global transcriptomic variation, with donor type‐associated differences observed in senescence‐associated genes, including *CDKN1A* (p21^CIP1^) and *CDKN2A* (p16^INK4a^/p19^ARF^). Together, these findings support an association between donor type and the observed transcriptomic and senescence‐associated differences, although the influence of donor age cannot be fully excluded. Moreover, senescent cells are heterogeneous in phenotype, and senescence‐associated gene sets such as the SenMayo capture a broad range of transcriptional pathways that overlap with inflammatory signaling and tissue injury. Therefore, the senescence‐associated transcriptional signals observed in donor liver tissue may partly reflect stress responses that intersect with, but are not specific to, cellular senescence. Such overlaps between senescence‐associated and inflammatory pathways have been described in the setting of liver aging and diseases, highlighting the complexity of defining senescence signatures (Karpova et al. 2026). In this context, guidelines for the definition and characterization of cellular senescence emphasize that senescence should not be inferred from a single marker or transcriptional signature alone, but rather assessed using multiple complementary features, including cell‐cycle arrest, SASP activity, DNA damage responses, and metabolic changes, while considering the tissue context (Ogrodnik et al. 2024). Future studies using single‐cell RNA‐sequencing, spatial transcriptomics, multiplexed immunostaining with auxiliary markers, or detection of cell‐type‐specific SASP are required to determine which cell populations contribute to these senescence‐associated signatures (Ogrodnik et al. 2024).

In conclusion, donor type reflects distinct transcriptional states in human donor livers, with senescence‐associated profiles reflecting donor type‐specific injury. Beyond its potential role as a marker of graft quality, injury‐induced senescence‐associated signaling may influence both graft function and recipient (patho)physiology, thereby positioning it as a promising modifiable senolytic or senomorphic target in liver transplantation.

## Author Contributions

Adam M. Thorne: concept, formal analysis, investigation, writing original draft. Sabrina Stimmeder: formal analysis, investigation, writing original draft. Kirill Ustyantsev: investigation, formal analysis. Niels Mulder: investigation. Laura Niedernhofer: writing review and editing. Ton Lisman: writing review and editing. Marco Demaria: writing review and editing. Eugene Berezikov: resources, writing review and editing, supervision. Folkert Kuipers: resources, writing review and editing, supervision. Vincent E. de Meijer: concept, methodology, resources, writing review and editing, supervision, project admin, funding.

## Funding

Vincent E. de Meijer has received institutional research grants from the Dutch Research Council (NWO; grant #09150161810030 and #09120242410037), from the Dutch Ministry of Economic Affairs (Health~Holland Public Private Partnership grant #PPP‐2019‐024 and #PPP‐2024‐010), and from the Dutch Society for Gastroenterology (NVGE #01‐2021), all outside the submitted work. Vincent E. de Meijer and Folkert Kuipers are supported by unrestricted grants from the Noaber Foundation (Lunteren, The Netherlands). Eugene Berezikov and Folkert Kuipers have received institutional research grants from the Dutch Research Council (NWO; grant #09120242410037).

## Ethics Statement

All donor samples and associated clinical data were prospectively collected as part of the TransplantLines cohort study and biobank (University Medical Center Groningen Medical Ethics Committee approval 2014/077, ClinicalTrials.gov identifier NCT03272841). Because only donor data and biological samples were analyzed, the study was exempt from additional institutional review. This study was conducted in accordance with the Declaration of Helsinki and the Declaration of Istanbul, and followed institutional ethical standards.

## Conflicts of Interest

Vincent E. de Meijer has received speaker's honoraria paid to the institution by Astellas, Chiesi, and XVIVO. Folkert Kuipers acts as consultant for Mirum with honorarium paid to the institution. Marco Demaria reports a relationship with Rubedo Life Sciences Inc., Oisin Biotechnologies, and Cleara biotech that includes board membership and equity or stocks. The other authors have nothing to disclose.

## Supporting information


**Supplementary Table 1:** Liver‐specific SASP‐associated panel. SASP, senescence‐associated secretory phenotype.
**Supplementary Table 2:** Available SenMayo gene transcripts in the external cohort.
**Supplementary Table 3:** Available liver‐specific SASP‐associated transcripts in the external cohort. SASP, senescence‐associated secretory phenotype.

**Supplementary Figure 1:** Immunohistochemical tissue assessment and gene expression of senescence markers. (A) Representative whole‐slide immunohistochemical image. Regions of interest were manually annotated to include relevant tissue while excluding artifacts. Annotated regions were subdivided into 1000 μm square tiles. (B) Square annotation tile. (C, D) Violin plots showing the percentage of (C) p16‐positive and (D) p21‐positive nuclei in individual ROI annotation tiles. DBD, donation after brain death; DCD, donation after circulatory death; ROI, region of interest.

**Supplementary Figure 2:** SenMayo genes associated with pathways in DBD and DCD donors. Circos plot mapping the SenMayo Gene Ontology pathways and their contributing genes in DBD (blue) and DCD (red) livers. DBD, donation after brain death; DCD, donation after circulatory death.

**Supplementary Figure 3:** Differential gene expression of liver‐relevant SASP‐associated injury panel. Volcano plot of transcripts of genes in the liver‐relevant SASP‐associated injury panel. Significant transcripts (*p* < 0.05, |log2FC > 1|) are highlighted in red for upregulated (enriched in DCD), blue for downregulated (enriched in DBD), and gray not significant. DBD, donation after brain death; DCD, donation after circulatory death; SASP, senescence‐associated secretory phenotype.

**Supplementary Figure 4:** Differential gene expression of all transcripts in the external RNA‐sequencing data. Volcano plot of all transcripts of the external cohort. Significant transcripts (*p* < 0.05, |log2FC > 1|) are highlighted in red for upregulated (enriched in DCD) and blue for downregulated (enriched in DBD). DBD, donation after brain death; DCD, donation after circulatory death.

## Data Availability

RNA sequencing data used for transcriptomics analysis are available through the European Nucleotide Archive (E‐MTAB‐17879). All other data are available in the main text or Supplementary Tables 1–3 and Supplementary Figures [Link], [Link], [Link], [Link].

## References

[acel70828-bib-0001] Ajoolabady, A. , D. Pratico , S. Bahijri , et al. 2025. “Hallmarks and Mechanisms of Cellular Senescence in Aging and Disease.” Cell Death Discovery 11, no. 1: 364. 10.1038/s41420-025-02655-x. PMC12322153 40759632

[acel70828-bib-0002] Braun, H. , B. M. Schmidt , M. Raiss , et al. 2012. “Cellular Senescence Limits Regenerative Capacity and Allograft Survival.” Journal of the American Society of Nephrology 23, no. 9: 1467–1473. 10.1681/ASN.2011100967. PMC3431409 22797186

[acel70828-bib-0003] Campisi, J. , and F. d'Adda di Fagagna . 2007. “Cellular Senescence: When Bad Things Happen to Good Cells.” Nature Reviews. Molecular Cell Biology 8, no. 9: 729–740. 10.1038/nrm2233. 17667954

[acel70828-bib-0004] Chen, C. , M. Zheng , H. Hou , et al. 2022. “Cellular Senescence in Ischemia/Reperfusion Injury.” Cell Death Discovery 8, no. 1: 420. 10.1038/s41420-022-01205-z. PMC9576687 36253355

[acel70828-bib-0005] Du, K. , D. S. Umbaugh , N. Ren , and A. M. Diehl . 2026. “Cellular Senescence in Liver Diseases: From Molecular Drivers to Therapeutic Targeting.” Journal of Hepatology 84, no. 1: 194–212. 10.1016/j.jhep.2025.08.021. PMC12538522 40882922

[acel70828-bib-0006] Eden, J. , P. C. Muller , C. Kuemmerli , et al. 2025. “Life Expectancy of Transplanted Livers: HOPE Against Aging?” Annals of Surgery 282, no. 5: 765–771. 10.1097/SLA.0000000000006883. PMC12513047 40772946

[acel70828-bib-0007] Eisenga, M. F. , A. W. Gomes‐Neto , M. van Londen , et al. 2018. “Rationale and Design of TransplantLines: A Prospective Cohort Study and Biobank of Solid Organ Transplant Recipients.” BMJ Open 8, no. 12: e024502. 10.1136/bmjopen-2018-024502. PMC6318532 30598488

[acel70828-bib-0008] Esser, H. , I. E. M. de Jong , F. M. Roos , et al. 2025. “Consensus Classification of Biliary Complications After Liver Transplantation: Guidelines From the BileducTx Meeting.” British Journal of Surgery 112, no. 5: znae321. 10.1093/bjs/znae321. PMC12046073 40313074

[acel70828-bib-0009] Esser, H. , A. M. Kilpatrick , T. Y. Man , et al. 2024. “Primary Cilia as a Targetable Node Between Biliary Injury, Senescence and Regeneration in Liver Transplantation.” Journal of Hepatology 81, no. 6: 1005–1022. 10.1016/j.jhep.2024.06.002. 38879173

[acel70828-bib-0010] European Association for the Study of the Liver . 2024. “EASL Clinical Practice Guidelines on Liver Transplantation.” Journal of Hepatology 81, no. 6: 1040–1086. 10.1016/j.jhep.2024.07.032. 39487043

[acel70828-bib-0011] Ferreira‐Gonzalez, S. , T. Y. Man , H. Esser , et al. 2022. “Senolytic Treatment Preserves Biliary Regenerative Capacity Lost Through Cellular Senescence During Cold Storage.” Science Translational Medicine 14, no. 674: eabj4375. 10.1126/scitranslmed.abj4375. 36475903

[acel70828-bib-0012] Ferreira‐Gonzalez, S. , T. Matsumoto , E. Hara , and S. J. Forbes . 2025. “Senescence, Aging and Disease Throughout the Gastrointestinal System.” Gastroenterology 169, no. 7: 1357–1379. 10.1053/j.gastro.2025.06.010. 40532827

[acel70828-bib-0013] Foley, J. W. , C. Zhu , P. Jolivet , et al. 2019. “Gene Expression Profiling of Single Cells From Archival Tissue With Laser‐Capture Microdissection and Smart‐3SEQ.” Genome Research 29, no. 11: 1816–1825. 10.1101/gr.234807.118. PMC6836736 31519740

[acel70828-bib-0014] Huang, W. , L. J. Hickson , A. Eirin , J. L. Kirkland , and L. O. Lerman . 2022. “Cellular Senescence: The Good, the Bad and the Unknown.” Nature Reviews. Nephrology 18, no. 10: 611–627. 10.1038/s41581-022-00601-z. PMC9362342 35922662

[acel70828-bib-0015] Iske, J. , T. Matsunaga , H. Zhou , and S. G. Tullius . 2021. “Donor and Recipient Age‐Mismatches: The Potential of Transferring Senescence.” Frontiers in Immunology 12: 671479. 10.3389/fimmu.2021.671479. PMC8113632 33995411

[acel70828-bib-0016] Iske, J. , M. J. Roesel , F. Martin , et al. 2024. “Transplanting Old Organs Promotes Senescence in Young Recipients.” American Journal of Transplantation 24, no. 3: 391–405. 10.1016/j.ajt.2023.10.013. PMC10922683 37913871

[acel70828-bib-0017] Iske, J. , M. Seyda , T. Heinbokel , et al. 2020. “Senolytics Prevent Mt‐DNA‐Induced Inflammation and Promote the Survival of Aged Organs Following Transplantation.” Nature Communications 11, no. 1: 4289. 10.1038/s41467-020-18039-x. PMC7453018 32855397

[acel70828-bib-0018] Karpova, A. , X. Li , C. W. Peng , et al. 2026. “Cellular Senescence in Human Liver Under Normal Aging and Cancer.” Cell Genomics 6, no. 2: 101133. 10.1016/j.xgen.2025.101133. PMC12903365 41576948

[acel70828-bib-0019] Kayumov, M. , Z. Song , F. Martin , et al. 2025. “The Promise of Organ Rejuvenation to Overcome the Shortage in Organ Transplantation.” Nature Communications 16, no. 1: 11259. 10.1038/s41467-025-66133-9. PMC12717245 41372118

[acel70828-bib-0020] Keslar, K. S. , Q. Liu , W. M. Baldwin , et al. 2025. “Normothermic Machine Perfusion Converges Transplant Induced Transcript Profiles in Low and High Risk DBD and DCD Livers.” Scientific Reports 15, no. 1: 34419. 10.1038/s41598-025-17448-6. PMC12491471 41039014

[acel70828-bib-0021] Kiourtis, C. , M. Terradas‐Terradas , L. M. Gee , et al. 2024. “Hepatocellular Senescence Induces Multi‐Organ Senescence and Dysfunction via TGFbeta.” Nature Cell Biology 26, no. 12: 2075–2083. 10.1038/s41556-024-01543-3. PMC11628396 39537753

[acel70828-bib-0022] Lascaris, B. , V. E. de Meijer , and R. J. Porte . 2022. “Normothermic Liver Machine Perfusion as a Dynamic Platform for Regenerative Purposes: What Does the Future Have in Store for Us?” Journal of Hepatology 77, no. 3: 825–836. 10.1016/j.jhep.2022.04.033. 35533801

[acel70828-bib-0023] Lau, A. , B. K. Kennedy , J. L. Kirkland , and S. G. Tullius . 2019. “Mixing Old and Young: Enhancing Rejuvenation and Accelerating Aging.” Journal of Clinical Investigation 129, no. 1: 4–11. 10.1172/JCI123946. PMC6307961 30601138

[acel70828-bib-0024] Lopez‐Otin, C. , M. A. Blasco , L. Partridge , M. Serrano , and G. Kroemer . 2013. “The Hallmarks of Aging.” Cell 153, no. 6: 1194–1217. 10.1016/j.cell.2013.05.039. PMC3836174 23746838

[acel70828-bib-0025] Mansfield, L. , V. Ramponi , K. Gupta , et al. 2024. “Emerging Insights in Senescence: Pathways From Preclinical Models to Therapeutic Innovations.” NPJ Aging 10, no. 1: 53. 10.1038/s41514-024-00181-1. PMC11584693 39578455

[acel70828-bib-0026] Monbaliu, D. , J. Pirenne , and D. Talbot . 2012. “Liver Transplantation Using Donation After Cardiac Death Donors.” Journal of Hepatology 56, no. 2: 474–485. 10.1016/j.jhep.2011.07.004. 21782762

[acel70828-bib-0027] Nehme, J. , L. Mesilmany , M. Varela‐Eirin , et al. 2024. “Converting Cell Death Into Senescence by PARP1 Inhibition Improves Recovery From Acute Oxidative Injury.” Nat Aging 4, no. 6: 771–782. 10.1038/s43587-024-00627-x. 38724734

[acel70828-bib-0028] Ogrodnik, M. , J. Carlos Acosta , P. D. Adams , et al. 2024. “Guidelines for Minimal Information on Cellular Senescence Experimentation In Vivo.” Cell 187, no. 16: 4150–4175. 10.1016/j.cell.2024.05.059. PMC11790242 39121846

[acel70828-bib-0029] Posthumus, A. M. , T. J. Knobbe , D. Kremer , et al. 2025. “TransplantLines, a Biobank and Cohort Study of Solid Organ Transplant Recipients and Donors.” European Journal of Epidemiology 40, no. 8: 969–979. 10.1007/s10654-025-01258-1. PMC12374880 40601244

[acel70828-bib-0030] Sanfeliu‐Redondo, D. , A. Gibert‐Ramos , and J. Gracia‐Sancho . 2024. “Cell Senescence in Liver Diseases: Pathological Mechanism and Theranostic Opportunity.” Nature Reviews. Gastroenterology & Hepatology 21, no. 7: 477–492. 10.1038/s41575-024-00913-4. 38485755

[acel70828-bib-0031] Saul, D. , R. L. Kosinsky , E. J. Atkinson , et al. 2022. “A New Gene Set Identifies Senescent Cells and Predicts Senescence‐Associated Pathways Across Tissues.” Nature Communications 13, no. 1: 4827. 10.1038/s41467-022-32552-1. PMC9381717 35974106

[acel70828-bib-0032] Shirini, K. , A. Al Ramlawi , A. Longchamp , C. Bhati , and R. P. H. Meier . 2026. “Effect of Donor‐Recipient Age Difference on Liver Transplant Outcomes.” Transplantation Direct 12, no. 3: e1906. 10.1097/TXD.0000000000001906. PMC12900182 41694481

[acel70828-bib-0033] Valdivieso, K. , T. Rozmaric , S. Victorelli , et al. 2026. “Transcription‐Independent Induction of Rapid‐Onset Senescence Is Integral to Healing.” Nature Cell Biology 28: 1281–1299. 10.1038/s41556-026-01948-2. PMC13279267 42209725

[acel70828-bib-0034] van Rijn, R. , N. Karimian , A. P. M. Matton , et al. 2017. “Dual Hypothermic Oxygenated Machine Perfusion in Liver Transplants Donated After Circulatory Death.” British Journal of Surgery 104, no. 7: 907–917. 10.1002/bjs.10515. PMC5484999 28394402

[acel70828-bib-0035] Wang, B. , J. Han , J. H. Elisseeff , and M. Demaria . 2024. “The Senescence‐Associated Secretory Phenotype and Its Physiological and Pathological Implications.” Nature Reviews. Molecular Cell Biology 25, no. 12: 958–978. 10.1038/s41580-024-00727-x. 38654098

[acel70828-bib-0036] Watts, R. P. , O. Thom , and J. F. Fraser . 2013. “Inflammatory Signalling Associated With Brain Dead Organ Donation: From Brain Injury to Brain Stem Death and Posttransplant Ischaemia Reperfusion Injury.” Journal of Transplant 2013: 521369. 10.1155/2013/521369. PMC3649190 23691272

