# Supplementary material for: Transcriptomic Characterization of Cellular Senescence in Human Donor Livers Before Transplantation
Source: Aging Cell. 2026 Nov 11;25(11):e70828. doi: 10.1111/acel.70828 (PMC13755069; doi:10.1111/acel.70828)

Supplementary Figure 1

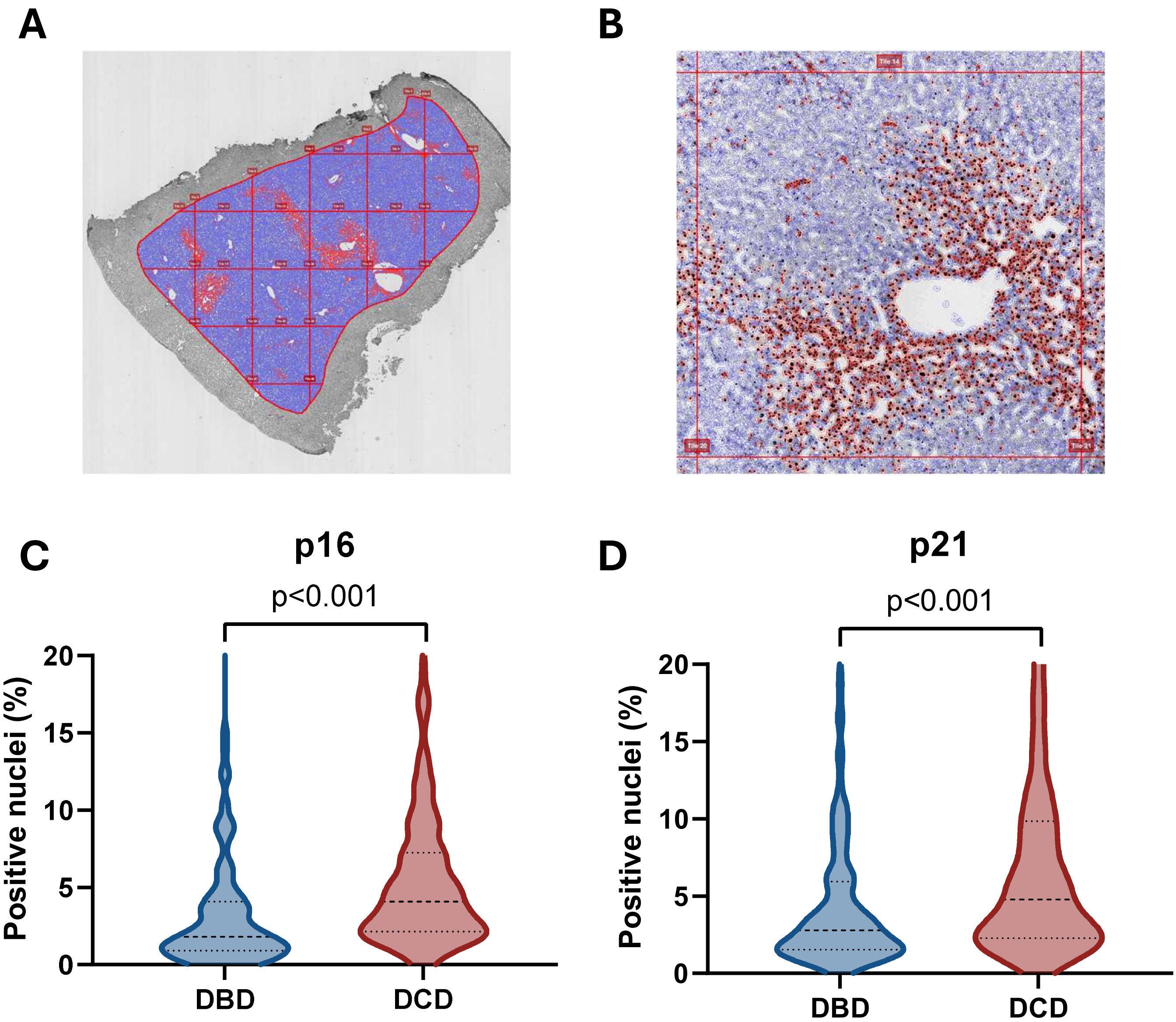

Supplement: Supplementary file 3 [file ACEL-25-e70828-s004.pdf]
