# Supplementary material for: Transcriptomic Characterization of Cellular Senescence in Human Donor Livers Before Transplantation
Source: Aging Cell. 2026 Nov 11;25(11):e70828. doi: 10.1111/acel.70828 (PMC13755069; doi:10.1111/acel.70828)
Supplement: Supplementary file 5 [file ACEL-25-e70828-s001.docx]

**SUPPLEMENTARY DIGITAL CONTENT**

**Transcriptomic Characterization of Cellular Senescence in Human Donor Livers before transplantation**

Adam M. Thorne^1,2*^, Sabrina Stimmeder^1,2,3*^, Kirill Ustyantsev^4^, Niels Mulder^5^, Laura Niedernhofer^6^, Ton Lisman^1^, Marco Demaria^4^, Eugene Berezikov^4^, Folkert Kuipers^4,5^, Vincent E. de Meijer^1,2#^

^1^ Department of Liver Transplantation and HPB Surgery, University of Groningen and University Medical Center Groningen, Groningen, the Netherlands

^2^ UMCG Comprehensive Transplant Center, Groningen, the Netherlands

^3^ Department of Surgery, Division of General, Visceral and Transplant Surgery, Medical University of Graz, Graz, Austria

^4^ European Research Institute for the Biology of Ageing (ERIBA), University of Groningen and University Medical Center Groningen, Groningen, the Netherlands

^5^ Department of Pediatrics, University of Groningen, University of Groningen and University Medical Center Groningen, Groningen, the Netherlands

^6^ Masonic Institute on the Biology of Aging and Metabolism, University of Minnesota, Minneapolis, MN, USA; Department of Biochemistry, Molecular Biology and Biophysics, University of Minnesota, Minneapolis, MN, USA

\# Corresponding author:

Vincent E. de Meijer, MD, PhD

University Medical Center Groningen, Department of Surgery, Section of Hepatobiliary Surgery and Liver Transplantation, Groningen, The Netherlands

Email: v.e.de.meijer@umcg.nl

**Supplementary Table 1. Liver-specific SASP-associated panel.**

**Gene**

ABCC2

ABCC3

ACTA2

ANGPTL4

CCL2

CDK2

CDK4

CDKN1A

CEBPA

CISD2

CTSB

CTSD

CX3CL1

CXCL1

CXCL8

DLAT

FCGR2B

FOXM1

FOXO3

FOXO4

GDF15

GLB1

H2AX

HSP90AA1

HSP90AB1

IGFBP2

IGFBP3

IGFBP4

IL1B

IL32

IL33

KDR

KLF2

MAP1LC3A

MAP1LC3B

MAPK1

MAPK3

MMP2

NFE2L2

NR1H4

SAA4

SERPINE1

SIRT1

SOD2

SPP1

SQSTM1

TGFB1

TGFB3

TP53BP1

VEGFA

YAP1

**Supplementary Table 2. Available SenMayo gene transcripts in the external cohort.**

**Gene**

AXL

C3

CCL1

CCL13

CCL16

CCL2

CCL20

CCL26

CCL3

CCL3L1

CCL4

CCL5

CCL7

CCL8

CD55

CD9

CSF1

CSF2

CSF2RB

CXCL1

CXCL10

CXCL12

CXCL16

CXCL2

CXCL3

CXCR2

FAS

HMGB1

ICAM1

ICAM3

IL10

IL13

IL15

IL18

IL1A

IL1B

IL2

IL32

IL6

IL6ST

IL7

IL8

ITGA2

LCP1

MIF

PECAM1

PLAU

PLAUR

SELPLG

SPP1

TNF

TNFRSF10C

TNFRSF11B

TNFRSF1A

TNFRSF1B

VEGFA

VEGFC

**Supplementary Table 3. Available liver-specific SASP-associated transcripts in the external cohort.**

**Gene**

CCL2

CDKN1A

CX3CL1

CXCL1

FCGR2B

IL1A

IL1B

IL32

IL8

MAPK1

MAPK3

SPP1

TGFB1

TGFB2

VEGFA
